# Targeted Therapy for Breast Cancer Prevention

**DOI:** 10.3389/fonc.2013.00250

**Published:** 2013-09-23

**Authors:** Petra den Hollander, Michelle I. Savage, Powel H. Brown

**Affiliations:** ^1^Department of Clinical Cancer Prevention, The University of Texas MD Anderson Cancer Center, Houston, TX, USA

**Keywords:** breast, cancer, prevention, therapy, TNBC

## Abstract

With a better understanding of the etiology of breast cancer, molecularly targeted drugs have been developed and are being testing for the treatment and prevention of breast cancer. Targeted drugs that inhibit the estrogen receptor (ER) or estrogen-activated pathways include the selective ER modulators (tamoxifen, raloxifene, and lasofoxifene) and aromatase inhibitors (AIs) (anastrozole, letrozole, and exemestane) have been tested in preclinical and clinical studies. Tamoxifen and raloxifene have been shown to reduce the risk of breast cancer and promising results of AIs in breast cancer trials, suggest that AIs might be even more effective in the prevention of ER-positive breast cancer. However, these agents only prevent ER-positive breast cancer. Therefore, current research is focused on identifying preventive therapies for other forms of breast cancer such as human epidermal growth factor receptor 2 (HER2)-positive and triple-negative breast cancer (TNBC, breast cancer that does express ER, progesterone receptor, or HER2). HER2-positive breast cancers are currently treated with anti-HER2 therapies including trastuzumab and lapatinib, and preclinical and clinical studies are now being conducted to test these drugs for the prevention of HER2-positive breast cancers. Several promising agents currently being tested in cancer prevention trials for the prevention of TNBC include poly(ADP-ribose) polymerase inhibitors, vitamin D, and rexinoids, both of which activate nuclear hormone receptors (the vitamin D and retinoid X receptors). This review discusses currently used breast cancer preventive drugs, and describes the progress of research striving to identify and develop more effective preventive agents for all forms of breast cancer.

## Introduction

Breast cancer is the most common cancer among women, with an estimated 232,340 new cases, in 2013 in the U.S. alone (1). This represents close to 30% of all estimated new cancer cases in women, and after a decline in the incidence rate, largely due to the reduction in the use of hormone replacement therapy (2, 3), the incidence rate has been stable for the past decade (1). Although there has been a steady decrease in breast cancer mortality since the early 90s (1), due largely to improvements in the early detection and treatment of breast tumors (4), in the U.S. approximately 40,000 women will die of breast cancer this year (1). Despite these positive reductions in mortality, preventing breast cancer prior to its development remains the most effective way to reduce mortality resulting from this disease. Recent clinical trials have now demonstrated that effective prevention is possible for some forms of breast cancer, and rapid advances being made during this genomic era are providing further understanding of breast cancer subtypes, laying the foundation for the development of preventive therapies for all forms of breast cancer.

Genomic profiling of breast cancer using expression arrays has proven useful for the elucidating different molecular forms of breast cancer. The analysis of gene expression patterns by Perou et al. (5), led to the discovery and identification of four distinct molecular subtypes of breast cancer with RNA expression profiles dividing the tumors into at least four subgroups (5). These subgroups are characterized by variations in overexpression, with the luminal subgroup highly expressing genes normally associated with breast luminal cells, the second subgroup expressing genes typically active in breast basal epithelial cells (basal-like subgroup), and the third subgroup overexpressing human epidermal growth factor receptor 2 (HER2 subgroup), which is associated with a unique set of genes. The fourth tumor subgroup consists of tumors that cluster with normal breast samples, and are classified as normal-like breast tumors. Follow-up analysis of a tumor set approximately twice the size as the original, demonstrated distinct differences in survival and treatment response between different molecular subtypes (6) (see Table 1). Luminal A tumors were associated with the longest overall survival, while HER2 and basal-like tumors were associated with decreased survival. Similar results were observed with time to recurrence (6). It is important to note that these tumors were obtained prior to the widespread use of anti-HER2 therapies, thus explaining the poor survival of the HER2 group. These findings have since been confirmed in a population-based study (7). However, the drivers for decreased overall and disease-free survival in women with basal-like tumors remain unclear. In 2007, Perou et al. extended their initial findings by identifying yet another molecular subtype, the claudin-low tumors, which underexpress genes involved in tight-junctions and cell–cell adhesion, including several Claudin genes and E-cadherin and high expression of endothelial markers (8). From a clinical perspective, these claudin-low tumors are associated with a poor prognosis (9). The basal and claudin-low molecular subtypes significantly overlap the clinical triple-negative breast cancers (TNBCs), which have low levels of ER, PR, and HER2 proteins. TNBCs exhibit a high level of molecular heterogeneity, are highly aggressive, and have proven challenging for the development of targeted therapeutic treatments, and for the development of effective preventive strategies.

**Table 1:** **Molecular subtypes of breast cancer**.

Molecular subtype	Gene expression	Survival
**Perou et al. (5), Sorlie et al. (6)**		
Luminal	High expression of genes normally expressed in breast luminal cells	Longest overall survival
Basal-like	High expression of genes normally expressed in breast basal cells	Shortest overall survival
HER2	Overexpression of human epidermal growth factor receptor 2 (HER2) and a unique gene set	Decreased overall survival
Claudin-low	High expression of genes involved in tight-junctions and cell-to-cell adhesion, including E-cadherin and several claudin genes	Decreased overall survival
**Lehmann et al. (10)**		
Immunomodulatory	Overexpression of cytokine signaling and antigen processing pathway genes	Reduced relapse-free survival compared to mesenchymal stem-like
Mesenchymal	Overexpression of cell motility and differentiation genes	Reduced relapse-free survival compared to basal-like 1, basal-like 2, mesenchymal stem-like, and immunomodulatory
Mesenchymal stem-like	Overexpression of cell motility and differentiation genes	Longest relapse-free survival
Luminal androgen receptor	Activation of the hormone signaling pathways	Shortest relapse-free survival
Basal-like 1	Overexpression of cell cycle and cell division genes	Intermediate relapse-free survival
Basal-like 2	Enhancement of the growth factor signaling pathways	Intermediate relapse-free survival

To further define the TNBC subtypes, Lehmann et al. analyzed the gene expression profiles of 21 breast cancer data sets, which included almost 600 TNBCs and identified six distinct subtypes: (1) immunomodulatory, (2) mesenchymal, (3) mesenchymal stem-like, (4) luminal androgen receptor, (5) basal-like 1, and (6) basal-like 2 (10). Stratification of breast cancer cell lines according to the same gene expression profiles, demonstrated that these six TNBC subtypes are uniquely sensitive to different drugs. Furthermore survival varies across each of these subtypes, with mesenchymal stem-like and luminal androgen receptor associated with the longest and shortest relapse-free survival, respectively (see Table 1). Overexpression of immune cell processing genes (e.g., cytokine signaling and antigen processing pathway genes) characterizes the immunomodulatory subtype, forming a rationale for developing preventive agents targeting the immune system, cytokines, and immune signal transduction pathways. The mesenchymal and mesenchymal stem-like subtypes exhibit enrichment for pathways involved with cell motility and cell differentiation, providing the foundation for studies of drugs targeting pathways regulating cell migration. The luminal androgen receptor subtype is ER-negative. However, estrogen and androgen hormone signaling pathways are activated in these tumors, supporting the use of anti-androgens and possibly anti-estrogens, for the treatment and prevention of these tumors. Upregulation of the cell cycle and cell division pathways defines basal-like subtype 1, suggesting the potential efficacy of anti-mitotic and DNA-damaging drugs, as well as poly(ADP-ribose) polymerase (PARP) inhibitors to treat and possibly prevent tumors of this type. The final subtype, basal-like subtype 2 is associated with enhancement of the growth factor signaling pathways, suggesting strategies targeting the epidermal growth factor receptor (EGFR), insulin-like growth factor 1 receptor (IGF1R), met proto-oncogene (MET), nerve growth factor (NGF), and Wnt/beta-catenin pathways, along with genes involved in gluconeogenesis and glycolysis, may be useful for the treatment and prevention of this group of tumors. The major challenge will be to determine the minimal number of drugs to combine to prevent all of these forms of cancer, and to select drugs that will be both effective and safe.

## Prevention of ER-Positive (Luminal) Breast Cancers

### Selective estrogen receptor modulators

**Figure 1:**
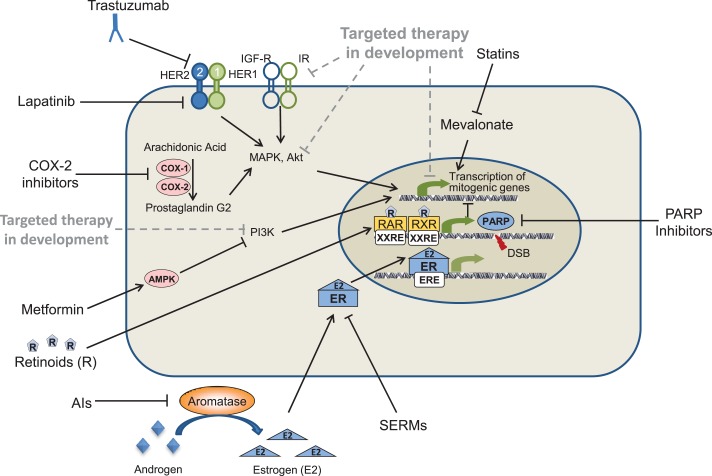
**Oncogenic pathways as molecular targets for the prevention of breast cancer**. Solid lines represent drugs and targets currently being used in the prevention of breast cancer; dotted lines represents drugs currently in development.

Estrogen and the estrogen receptors (ERs) are key regulators in the progression of breast cancer, as well as other hormonally stimulated cancers. For this reason, drugs targeting ER, known as selective estrogen receptor modulators (SERMs), were developed and have been used for decades to suppress the estrogen signaling pathway in women with breast cancer (Figure 1). Tamoxifen, the first SERM to be approved for the treatment of metastatic breast cancer, has been shown to have antagonistic effects in breast, while acting as an agonist in other tissues. Tamoxifen is routinely used to treat all stages of breast cancer. Adjuvant breast cancer trials have demonstrated that tamoxifen reduces both breast cancer recurrence and contra-lateral breast cancer by approximately 40–50% in women with early breast cancer (11). The positive results from these trials opened the door to test the preventive effects of tamoxifen in women without breast cancer. Four major Phase III breast cancer prevention trials have now demonstrated the efficacy of tamoxifen in women at high risk for developing breast cancer. Across these studies, tamoxifen reduced overall breast cancer incidence between 16 and 49% (12), and ER-positive breast cancer incidence between 31 and 69% (see Table 2).

**Table 2:** **Selective estrogen receptor modulator, AI, and HER2 breast cancer prevention clinical trials**.

Trial	Study design	Patient characteristics	Results
**SELECTIVE ESTROGEN RECEPTOR MODULATORS (SERMs)**			
**Tamoxifen**
Royal Marsden trial (13)	Tamoxifen vs. placebo	2,494 High-risk women	Reductions in all BC (16%) and ER-positive BC (39%)
NSABP-(BCPT)-P-1 (14)	Tamoxifen vs. placebo	13,388 High-risk women	49% Reduction in all BC
Italian trial (15)	Tamoxifen vs. placebo	5,408 Normal-risk women with hysterectomy	Reductions in BC (16%) and ER-positive BC (69%)
IBIS-I (16)	Tamoxifen vs. placebo	7,154 High-risk women	Reductions in all BC (27%) and ER-positive BC (31%)
**Raloxifene**
MORE (17)	Raloxifene (60 or 120 mg) vs. placebo	7,705 Normal-risk women with osteoporosis	Reductions in all BC (65%) and ER-positive BC (90%)
CORE (18)	Extension of MORE trial	5,213 Women from MORE trial	Reductions in all BC (50%) and ER-positive BC (66%)
RUTH (19)	Raloxifene vs. placebo	10,101 Postmenopausal women with coronary heart disease	Reductions in all BC (44%) and ER-positive BC (55%)
**Tamoxifen vs. raloxifene**
STAR (20)	Tamoxifen vs. raloxifene	19,747 High-risk women	5-years: raloxifene and tamoxifen equally effective for preventing progression to breast cancer
			81 months: raloxifene is 75% effective as tamoxifen
**Lasofoxifene**
PEARL (21)	Lasofoxifene vs. placebo	8,556 Women with osteoporosis	Reductions in all BC (79%) and ER-positive BC (81%)
**AROMATASE INHIBITORS (AIs)**			
IBIS-II – DCIS (12, 22)	Tamoxifen vs. anastrozole	4,000 Women with DCIS	Anticipated in 2 years
NSABP B-35	Tamoxifen vs. anastrozole	3,104 Women with ER-positive DCIS	Anticipated in 2 years
NCIC-MAP.3 (23)	Exemestane vs. placebo	4,560 Postmenopausal high-risk women	Reductions in all BC (65%) and ER-positive BC (75%)
IBIS-II (24)	Anastrozole vs. placebo	6,000 Postmenopausal high-risk women	Anticipated in 3 years
**HER2 INHIBITORS**			
**Trastuzumab**
Kuerer et al. (25)	Trastuzumab vs. placebo	24 Women with HER2-positive DCIS	No histologic evidence of response; increased ADCC in 100% of patients
Decensi et al. (26)	Lapatinib vs. placebo	60 Women with early HER2-positive cancer	Reduction in proliferation in early cancer and pre-cancer
Brown et al. (27)	Lapatinib vs. placebo	60 Women with EGFR or HER2-positive DCIS	Ongoing: endpoint alteration in proliferation

ADCC, antibody-dependent cellular cytotoxicity; BC, breast cancer; CORE, continued outcomes of raloxifene evaluation; DCIS, ductal carcinoma *in situ*; ER, estrogen receptor; HER2, human epidermal growth factor receptor 2; IHC, immunohistochemical staining; IBIS, Italian, Italian randomized tamoxifen prevention trial; MORE, multiple outcomes of raloxifene evaluation; NCIC CTG MAP.3, or NCIC-MAP.3, the National Institute of Canada Clinical Trials Group Mammary Prevention.3 trial; NSABP B-35, National Surgical Adjuvant Breast and Bowel Project B-35; NSABP-P-1, NSABP Breast Cancer Prevention Trial (BCPT) P-1; NSABP-P2, NSABP Study of Tamoxifen and Raloxifene (STAR) P2; PEARL, postmenopausal evaluation and risk reduction with lasofoxifene trial; RUTH, raloxifene use for the heart trial; Royal Marsden, Royal Marsden tamoxifen prevention trial.

The first of the breast cancer prevention trials to be conducted was the Royal Marsden Tamoxifen Breast Cancer Prevention Trial, which was conducted from 1986 to 1996 (13, 28, 29). This trial found a non-significant 39% reduction during the 8-year treatment period, but was significantly lower in the post-treatment period in ER-positive breast cancer incidence (13). There was no reduction in ER-negative breast cancer incidence. The second, and largest, clinical prevention trial testing tamoxifen was the National Surgical Adjuvant Breast and Bowel Project (NSABP) Breast Cancer Prevention Trial (BCPT) P-1 (14, 30). This trial recruited over 13,000 women at increased risk for invasive breast cancer to study the effects of 5 years of tamoxifen treatment in pre- and post-menopausal women. The results showed a significant 49% reduction in overall incidence of invasive breast cancer, and a 69% reduction in ER-positive breast cancers. There was not only reduction in the incidence of breast cancer in higher risk women without breast cancer, but an impressive reduction in women with a history of lobular carcinoma *in situ* or atypical hyperplasia of 56 and 86% respectively. These results formed the foundation for FDA approval of tamoxifen for the prevention of breast cancer in high-risk pre- and post-menopausal women in 1998. The results of the NSABP P-1 trial also identified tamoxifen-associated toxicities, including increased risk of thromboembolisms, endometrial cancer, hot flushes, vaginal symptoms, and cataracts (14, 30).

The Italian breast cancer prevention trial, the third to test the preventive effects of tamoxifen in breast cancer, included 5,408 women at normal risk for breast cancer. In order to avoid the increased incidence of endometrial cancers associated with tamoxifen use, only women who had previously undergone a hysterectomy were enrolled (15, 31). Although many of the women enrolled in this study were on hormone replacement therapy, which has since been shown to increase risk of breast cancer, a reduction of 20% in invasive breast cancer incidence was still observed.

The fourth breast cancer prevention clinical trial testing tamoxifen for the prevention of breast cancer was the International Breast Cancer Intervention Study I (IBIS-I), in which women at high-risk were treated with tamoxifen for 5 years (32). Tamoxifen reduced the occurrence of ER-positive breast cancers by 48%, and the long-term follow-up of this trial suggested a persistent benefit after stopping treatment for at least 10 years, demonstrating a 31% reduction in invasive ER-positive breast cancer after long-term follow-up (16). Another encouraging finding identified during the follow-up was a reduction in the negative side effects of tamoxifen after stopping the drug.

While each of the four trials demonstrated effective prevention of many ER-positive breast cancers with tamoxifen treatment, no reduction in the progression of ER-negative tumors was observed in any of these trials. All four trials reported long-term side-effects associated with tamoxifen treatment, including hot-flashes, night sweats, and vaginal symptoms, as well as more severe side effects, including increased risk of endometrial cancer and thromboembolism. Due to these tamoxifen-related side effects, focus shifted toward the development of less toxic second generation SERMs.

The first trial investigating a SERM other than tamoxifen for breast cancer prevention was the Multiple Outcomes of Raloxifene Evaluation (MORE) trial that tested the effect of raloxifene on bone fractures in postmenopausal women with osteoporosis, and also tested its effect on several other endpoints, including breast cancer (see Table 2). The trial demonstrated a 76% reduction in invasive breast cancer incidence and a 90% reduction of ER-positive breast cancer incidence after 3 years of treatment (17). These impressive results led to the development of two additional clinical cancer prevention trials testing the effectiveness of raloxifene in the prevention of invasive breast cancer. These trials, the Continued Outcomes of Raloxifene Evaluation (CORE) trial and the Raloxifene Use for the Heart (RUTH) trials, were conducted concurrently by two different groups. The CORE trial was an extension of the MORE trial, and confirmed that long-term treatment with raloxifene reduces the incidence rate of invasive ER-positive breast cancers, but has no preventive effect on ER-negative breast cancers (18). The long-term follow-up of women on either 4 or 8 years of raloxifene treatment showed decreases of 31 and 59% in invasive breast cancer, respectively (33). This clearly indicates that extended treatment with raloxifene prevents ER-positive breast cancer. The RUTH trial, on the other hand, was specifically designed to evaluate the effects of 5 years of raloxifene treatment on the incidence rates of coronary events and invasive breast cancer. A 44% reduction of overall invasive breast cancer incidence and a 55% of ER-positive breast cancer incidence was observed. These results were similar to the MORE and CORE trails (19, 34). Another important observation was that no statistically significant increase in incidence of endometrial cancer was observed in any of the three raloxifene trials.

Based on the collective results of the tamoxifen and raloxifene clinical prevention trials, the NSABP Study of Tamoxifen and Raloxifene (STAR) trial was developed to compare the effectiveness of 5 years of treatment with these two drugs in decreasing risk of breast cancer in high-risk postmenopausal women (see Table 2) (20). The initial results showed that tamoxifen and raloxifene were equally effective in their ability to decrease risk of breast cancer, with both reducing agents risk by approximately 50% (20). However, the study also identified differences in toxicities between the two arms, with patients receiving raloxifene reporting fewer side-effects and having fewer cases of blood clots and uterine cancers. The long-term follow-up of the STAR trial revealed that raloxifene was less effective for the prevention of invasive breast cancer, retaining only 76% of its long-term preventive effects compared to tamoxifen (35). Thus, the cancer preventive effect of raloxifene is not as persistent as that of tamoxifen. However, a major potential advantage of raloxifene is that it has fewer side-effects (decreased incidence of uterine cancers and thromboembolisms in raloxifene-treated women). Therefore, postmenopausal women and their doctors have a choice: they can choose the most effective preventive SERM, tamoxifen, and accept its toxicities, or they can choose the slightly less effective, but more tolerable SERM, raloxifene. In 2007, based upon the early findings in the Star trial, raloxifene received FDA approval for preventive treatment of postmenopausal women with osteoporosis or at high risk for invasive breast cancer.

More recently, the third generation SERM lasofoxifene has been tested as a breast cancer preventive drug (see Table 2). A Phase III clinical trial demonstrated the preventive efficacy of lasofoxifene in postmenopausal women with osteoporosis. The Postmenopausal Evaluation and Risk Reduction with Lasofoxifene (PEARL) trial, in which women were treated with lasofoxifene for 5 years, reported an 81% reduction in ER-positive breast cancer incidence. In addition, lasofoxifene treatment was associated with decreased toxicity compared to both tamoxifen and raloxifene (21). Despite this extremely high level of efficacy, lasofoxifene has not yet been FDA-approved for a breast cancer prevention indication. Thus, tamoxifen and raloxifene currently remain the only SERMs approved by the FDA for the prevention of breast cancer.

## Aromatase Inhibitors

Aromatase inhibitors (AIs) represent an alternative strategy to prevent ER-dependent breast cancers. AIs such as letrozole, anastrozole, and exemestane, block the biosynthesis of estrogen from androgens through the inhibition of the aromatase enzyme, resulting in drastic reductions in the circulating estrogen levels of serum, tissue, and tumor cells (36) (Figure 1). The aromatase enzyme is present in fat, stromal, and muscle cells, but is also expressed in breast tumors (37). In adjuvant breast cancer trials, AIs have proven to be an effective treatment strategy in premenopausal women with ER-positive breast cancer, even more effective than tamoxifen. As with tamoxifen, the cancer preventive potential of AIs was suggested by results of early breast cancer trials which showed a reduction in contralateral breast incidence. Several clinical trials demonstrated increased time to recurrence and improved efficacy in the prevention of a second primary breast cancers following treatment with AIs as compared to tamoxifen (23, 38). In addition, AI treatment is not associated with increased thromboembolic events and uterine cancers, although increased risk of bone fractures has also been observed (39). The National Cancer Institute of Canada Clinical Trials Group MA.17 (NCIC CTG MA.17) trial demonstrated that switching to the AI letrozole after 5 years of tamoxifen reduced the risk of contra-lateral breast cancer in patients diagnosed with ER-positive breast cancer (40) The largest of the trials investigating the efficacy of an AI as an adjuvant therapy was the Arimidex, Tamoxifen Alone or in Combination (ATAC) trial. The long-term follow-up of this trial demonstrated that AI therapy with anastrozole produced more persistent and effective protection against contralateral ER-positive breast cancer incidence than tamoxifen (41).

Two cancer prevention trials are currently ongoing in women with ductal carcinoma *in situ* (DCIS), comparing the AI anastrozole and tamoxifen, the NSABP B-35 and IBIS-II (DCIS) trials (see Table 2). Results from these trials are expected to be released in 1–2 years. In both of these trials women with ER-positive DCIS are being treated with the AI anastrozole or tamoxifen for 5 years. Both the NSABP B-35 and IBIS-II (DCIS) trials have completed accrual and results are anticipated in the near future. The primary endpoint of both trials is the incidence of invasive breast cancer, with important secondary endpoints including incidence of contralateral breast cancer and toxicity.

Based on the results of the adjuvant AI therapy trials, several large clinical cancer prevention trials were developed to test the effectiveness of AIs in preventing breast cancer in high-risk women without breast cancer. Two Phase III prevention trials are currently being conducted testing AIs in postmenopausal women, the MAP.3 trial and the IBIS-II prevention trials (see Table 2) (12, 22, 42). Results of the MAP.3 trial comparing the AI exemestane with placebo in high-risk women without breast cancer have been reported and show a 74% reduction in invasive ER-positive breast cancer incidence, associated with no increased in incidence of osteoporosis or endometrial cancers, following treatment with the AI exemestane. However, this trial had a short follow-up period when these results were reported. More significant toxic effects may be observed with a longer follow-up. For these reasons, exemestane is not yet FDA-approved for the prevention of breast cancer.

The early results of the MAP.3 trial suggest AIs will be highly effective for the prevention of ER-positive breast cancer. However, neither SERMs nor AIs prevent ER-negative breast cancer. These observations indicate that to prevent ER-negative breast cancers it will be necessary to target molecules critical for the growth and progression of ER-negative tumors.

## HER2 Inhibitors

Human epidermal growth factor receptor 2 is a member of the EGFR family of receptor tyrosine kinases. Upon phosphorylation of MAPK, EGFR signaling regulates the transportation of mitogenic signals across the cell membrane via a signaling cascade. Once intracellular, these signals induce proliferation and inhibit cell death; thus, misregulation of this pathway results in uncontrolled growth and inhibition of apoptosis. Overexpression of growth factor receptors has been identified in many different cancers, with HER2 overexpression present in 20–25% of breast cancers (43, 44). By targeting HER2 through different molecular mechanisms, EGFR inhibitors, and particularly HER2 inhibitors (including trastuzumab and the dual EGFR/HER2 inhibitor lapatinib), inhibit tumor growth, and induce apoptosis. The humanized HER2 antibody trastuzumab, targets the extracellular domain of HER2, while lapatinib inhibits the kinase activity of HER2 and EGFR (Figure 1). Both trastuzumab (44) and lapatinib (43) have been shown to be effective in the adjuvant setting for women with HER2-positive breast cancer. Research is now focused on determining whether HER2-positive breast cancers can be prevented by treating patients earlier at the stage of non-invasive breast cancer. Our group has shown that HER2-transgenic mice treated with the HER2/EGFR dual kinase inhibitor lapatinib (45) or the EGFR inhibitor gefitinib (46) have delayed development of HER2-positive mammary tumors. These studies demonstrated that treatment with the lapatinib also inhibits the development of mammary gland pre-malignant lesions in these mice (45).

Due to these positive adjuvant clinical trial and preclinical study results demonstrating delay of HER2-positive tumors following treatment with HER2 inhibitors, clinical cancer prevention trials have been developed to test trastuzumab or lapatinib in women with HER2-positive DCIS lesions. A Phase II trial testing a single pre-operative dose of trastuzumab in women with HER2-positive DCIS demonstrated an immunologic response that was not associated with either a pathologic or proliferation-related response (see Table 2) (25). The ongoing NSABP B-43 trial is testing the effects of radiation alone or in combination with two doses of trastuzumab (after surgical excision of the DCIS) on ipsilateral incidence of recurrent DCIS, invasive breast cancer or skin cancer. The results of a Phase II trial recently testing lapatinib for the treatment of HER2-positive breast cancer (early-stage invasive breast cancer and DCIS) showed that lapatinib (at 1,500 mg/day) decreased breast cancer cell proliferation in ER-negative tumors and in DCIS and ductal hyperplasia lesions (26). Another similar trial in women with HER2-positive or EGFR-positive DCIS breast cancer testing the effect of a lower dose of lapatinib (1,000 mg/day) is currently ongoing.

To date, the results from the preclinical and early clinical cancer prevention trials studying the effectiveness of EGFR inhibitors as viable preventive strategies for women with HER2-positive breast cancer are very promising. However, breast cancers that do not express ER, PR, or HER2, will not benefit from these targeted treatments.

In addition to HER2 inhibitors, HER2 peptide vaccines are being studied as therapeutic agents to induce immune responses to HER2-positive breast cancers. Such HER2 vaccines may in the future be most useful for the prevention of HER2-positive breast cancers. HER2 antibodies have been shown to be present in prediagnostic breast cancer sera (47). This observation supports the development of anti-HER2 vaccine approaches using HER2 peptides as immunogens. Responses to these HER2 peptide vaccines are restricted to specific major histocompatibility complex (MHC) classes: Class I (E75, GP2) and II (AE37) peptides simulate CD8- and CD4-positive T cells, respectively, and have been shown to induce an antitumor response (48). Results from Phase I and II clinical trials using these HER2 peptide vaccines have demonstrated significant immunologic *ex vivo* and *in vivo* responses (49, 50), and improved disease-free survival (particularly in patients with low-HER2 expression) persisting over time (51). In addition, all of these studies have shown that anti-HER2 vaccination has minimal toxicity and is easily tolerated by women with prior breast cancer. The first Phase III clinical trial investigating the efficacy of an anti-HER2 vaccine (E37) given as adjuvant therapy to women with early-stage node-positive breast cancer (the Prevention of Recurrence in Early-Stage, Node-Positive Breast Cancer with Low to Intermediate HER2 Expression with NeuVax Treatment, or PRESENT, trial) is currently ongoing. Future studies will focus on testing whether these peptides will be useful in high-risk women for the prevention of HER2-positive breast cancer.

## Development of Preventive Agents for Triple-Negative Breast Cancer

Triple-negative breast cancers represent 15–20% of all breast cancers, and are defined by a lack of ER, PR, and HER2 expression, resulting in limited treatment options. TNBCs are more aggressive, affect younger women, and are higher in incidence among women of African descent. In addition, these breast cancers have demonstrated both a higher rate of recurrence and a worse clinical outcome as compared to the other subtypes of breast cancer. Due to the lack of well-defined clinical targets, standard chemotherapy is currently the only treatment option for women with TNBC, and there are no available preventive drugs.

Recently, six distinct TNBC subgroups were identified through RNA expression profiling analyses, as mentioned in the introduction (10). Other studies have similarly identified a set of kinase gene expression profiles that divide ER-negative breast cancers into four distinct subtypes, including the cell cycle regulatory, S6 kinase, immunomodulatory, and MAPK clusters (52). In addition, survival analyses have shown that the subtype driven by the S6 kinase pathway carries the worst prognosis of the four ER-negative breast cancer subtypes, and emphasizes the importance of identifying druggable targets specific for each subtype of breast cancer.

## Retinoids

Retinoids are particularly promising drugs for the prevention of TNBC. These molecules, which are derivatives of vitamin A, bind retinoic acid receptors (RARs), and affect transcription factors that regulate gene expression to control development, differentiation, and homeostasis (53) (Figure 1). Studies by our group and others have demonstrated that retinoids are effective agents for the prevention of ER-negative breast cancer in animal models (54–58).

Early preclinical studies in rats indicated a correlation between vitamin A blood levels and changes in the epithelial tissue from a stratified keratinizing to a normal epithelium in several organs, which could be reversed back to stratified keratinized epithelium by restoring normal vitamin A (59). Further analysis in animals has since established the ability of retinoids to prevent cancer (60), demonstrating retinoid-mediated prevention of mammary carcinogenesis in rats after chemical carcinogen exposure, and in ER-negative mouse models (54).

A chemoprevention trial focused on the prevention of second primary tumors of the head and neck was one of the first clinical trials to demonstrate the effectiveness of retinoids for the prevention of cancer in humans (61). The retinoid 13-*cis*-retinoic acid was shown to reduce the incidence of second primary tumors; however, it has not been used clinically for prevention because of its toxicity. Similar toxicity has been observed with another retinoid, 9-*cis*-retinoic acid (62, 63). Treatment with the synthetic retinoid fenretinide (4HPR) for 5 years in a Phase III clinical trial in women with previous early stage breast cancer demonstrated no overall breast cancer preventive effect, but suggested a beneficial effect in premenopausal women (64). Longer follow-up after 15 years confirmed no overall breast cancer preventive effect, but showed a statistically significant 38% reduction in second primary breast cancers in premenopausal women (65). Fenretinide treatment was also associated with reduced incidence of ovarian cancer in women taking fenretinide; however, this protective effect was not apparent after stopping treatment (66).

Other synthetic retinoids, known as rexinoids, have been developed, which specifically bind the retinoid X receptor (RXR). These RXR ligands retain the cancer preventive activity of retinoids, but have much less toxicity. Extensive testing of rexinoids, including bexarotene and the more RXR-selective drug LG100268, has demonstrated a preventive effect with reduced toxicity compared to retinoids in animal models [e.g., the MMTV-ErbB2 transgenic and C3(1)-SV40 T-antigen models] (55, 56). More recently, it has been shown that the combined treatment with rexinoids and anti-estrogen SERMs is more effective in preventing mammary tumors than treatment with either agent alone (67, 68).

The positive preclinical rexinoid results supported the development of a Phase II clinical trial testing the effect of 4 weeks of bexarotene treatment in women at high-risk for breast cancer. Biomarker analysis from this study demonstrated that bexarotene treatment caused a non-significant reduction in the proliferation marker Ki67, and a significant reduction of Cyclin D1 expression in postmenopausal women (27). However, bexarotene was associated with toxicities (skin rash and hypertriglyceridemia), which may limit its clinical use. The rexinoid LG100268 is even more effective in the prevention of mammary tumors than bexarotene and has significantly less toxicity, thus LG100268 is a promising candidate for ER-negative breast cancer prevention in the future (see Table 3).

**Table 3:** **Select additional preclinical and clinical studies of novel agents for breast cancer prevention**.

Trial/experiment	Study design	Treatment group characteristics	Results/primary endpoint(s)
**EGFR INHIBITORS**			
Chan et al. (69)	Gefitinib vs. placebo	Transplant of DCIS tissue in immuno-suppressed mice	56% Reduction in proliferation, measured by Ki67
Lu et al. (46)	Gefitinib (low and high dose) vs. placebo	MMTV-Erb2 mice	High dose gefitinib showed a delay in ER-negative tumor development
Piechocki et al. (70)	Gefitinib vs. placebo	MMTV-Erb2 mice	Reduction in number and size of tumors
Strecker et al. (45)	Lapatinib (low and high dose) vs. placebo	MMTV-Erb2 mice	High dose lapatinib showed a delay in ER-negative tumor development
**REXINOID**			
Li et al. (57)	LG100268 (low and high dose) vs. placebo	MMTV-Erb2 mice	Low dose: delay in ER-negative tumor development
			High dose: prevented ER-negative tumor development in 90% of mice
**COX-2 INHIBITORS**			
Fabian et al. NCT00056082	Celecoxib vs. placebo	110 Premenopausal women at high-risk for ER-negative BC	Proliferation: Ki67 IHC staining
Arun et al. N01-CA-9757	Exemestane ± celecoxib	44 Pre- and post-menopausal high-risk women	Proliferation: Ki67 IHC staining
Wong et al. NCI-04-0044	Exemestane ± celecoxib	72 Postmenopausal high-risk women	Mammographic breast density
**METFORMIN**			
Anisimov et al. (72)	Metformin vs. placebo	MMTV-Erb2 mice	Delay in ER-negative tumor development
**MTOR INHIBITORS**			
Torres-Arzayus et al. (73)	Everolimus vs. placebo	AIB Mice	Reversion of pre-malignant phenotype
Kim et al. (74)	Rapamycin vs. vehicle	Benign, pre-malignant, and breast cancer cell lines	Most effective in benign and pre-malignant cells
Mercier et al. (75)	Rapamycin vs. vehicle	Cav-1 knockout mice	Tumor growth inhibition; decreased stromal content
**IGF-R INHIBITORS**			
Litzenburger et al. (76)	BMS-754807 vs. placebo	MCF10A	Growth inhibition in a pre-malignant cell line transformed by IGF1R

BC, breast cancer; DCIS, ductal carcinoma *in situ*; ER, estrogen receptor; IHC, immunohistochemical staining.

## COX-2 Inhibitors

Some of the most promising cancer preventive drugs are the non-steroidal anti-inflammatory drugs, which specifically inhibit one or both of the cyclooxygenase (COX)-1 and COX-2 enzymes (Figure 1). While COX-1 is present in most tissues and overexpressed in a variety of cancers, COX-2 expression is induced by mitogenic signals and is primarily localized at sites of inflammation (77). A polyp prevention study conducted by Steinbach et al. showed that patients with familial adenomatous polyposis (FAP) treated with the COX-2 inhibitor celecoxib exhibited a significant regression of colorectal adenomas (78). These results led to FDA approval of celecoxib for the reduction of colonic polyps in patients with FAP. Unfortunately, although COX-2 inhibitors have proven effective drugs to prevent colonic polyp formation, several large polyp prevention studies identified rare but potentially severe cardiovascular toxicities associated with COX-2 inhibitors. These Phase III trials included the Adenomatous Polyp Prevention on Vioxx (APPROVe) trial (79), which studied the COX-2 inhibitor rofecoxib, and the Prevention of Colorectal Sporadic Adenomatous Polyps (PreSAP) (80) and Adenoma Prevention with Celecoxib (APC) (81) trials, which both studied the COX-2 inhibitor celecoxib.

The COX-2 inhibitor celecoxib has also been the focus of studies investigating its effectiveness in the prevention of ER-negative breast cancer. Celecoxib has been shown to significantly delay the onset of tumor formation in MMTV-erbB2 transgenic mice, which develop primarily ER-negative tumors (82). This observation is particularly relevant for the prevention of both ER-negative and TNBCs.

Following these positive preclinical results, several early phase breast cancer prevention clinical trials testing COX-2 inhibitors have been conducted (83, 84). Unfortunately, due to the increased risk of heart attacks reported in the polyp prevention trials, the FDA halted ongoing COX-2 trials, including several Phase II breast cancer prevention studies. However, since COX-2 has been shown to play an important role in multiple cancer types and COX-2 inhibition is effective in preventing ER-negative mammary tumors in mice, it is likely that research will continue to focus on the development of safer and more effective agents targeting the COX-2 pathway.

## Metformin

Metformin is the most frequently used drug for the treatment of type-2 diabetes, and has recently been investigated as a cancer prevention drug. A meta-analysis of epidemiologic studies recently confirmed an association between diabetes and increased risk of breast cancer (most apparent in postmenopausal patients) (85).

Metformin reduces glucose levels, resulting in the reduction of insulin levels (86), and treatment with metformin has been shown to result in the inhibition of breast cancer cell growth *in vitro* (87). Therefore, it is possible that decreased insulin levels may reduce the activation of pathways involved with cell growth, thereby reducing tumorigenesis. Metformin has also been shown to activate AMP-activated protein kinase (AMPK) (Figure 1), which recent studies suggest may overcome resistance to HER2 inhibitors (88). Metformin has also been shown to slow the growth of mammary tumors in MMTV-erbB2 transgenic mice (see Table 2) (72, 89).

Several early phase breast cancer clinical trials testing the effects of metformin on breast tissue biomarkers reported reductions in cell proliferation following treatment with metformin in women with operable invasive or early-stage breast cancer (see Table 4). Each of these studies measured proliferation by Ki67 staining in breast tumors (90–93). The results of clinical trials currently being conducted, including a number of Phase II trials and one Phase III trial, are anticipated in the upcoming years. Of these, the Phase III NCIC-MA.32 trial is of particular interest, and will examine the effect of metformin on invasive disease-free survival, overall survival, and contralateral breast cancer incidence in women diagnosed with early stage breast cancer. The results reported at the conclusion of these studies will further define the breast cancer preventive activity of metformin, and determine its relevance as an effective strategy for the prevention of ER-negative breast cancer.

**Table 4:** **Select metformin breast cancer prevention studies (completed or with preliminary results)**.

Trial	Study design	Patient characteristics	Results/primary endpoint(s)
**TRIALS WITH PUBLISHED RESULTS**			
Hadad et al. (91)	Metformin vs. non-metformin	55 Non-diabetic women with operable invasive breast cancer	Reduction in Ki67 staining in metformin pilot (5%) and metformin study (3.4%) groups
Bonanni et al. (92)	Metformin vs. placebo	200 Non-diabetic women with operable invasive breast cancer	Altered Ki67 staining overall (4.0%), in HOMA^a^ ≤ 2.8 (11.1%) and HOMA^a^ > 2.8 (−10.5%), and modified metformin effects in luminal B tumors (as per HOMA index
Goodwin et al. (90), Niraula et al. (93)	Metformin	39 Women under the age of 70 with untreated, early-stage breast cancer	2.97% Reduction in Ki67 staining (±9.78%), 0.49% increase in TUNEL staining (±1.0%), and patient toleration of drug
**ONGOING TRIALS (NO RESULTS PUBLISHED TO DATE)**			
Goodwin et al. Phase II NCT01310231	Metformin vs. placebo	78 Women with invasive breast cancer diagnosed within the past year	Progression-free survival (PFS)
Patterson et al. NCT01302379	Metformin vs. placebo in lifestyle intervention and standard dietary arms	340 Women with stage I–III breast cancer diagnosed within the past 5 years	Breast cancer survival biomarker levels
Hershman et al. Phase II NCT00930579	Metformin	35 Women with early invasive breast cancer or DCIS	Measurement of effects on AMPK/mTOR signaling and fasting serum insulin levels
Harris et al. Phase III NCT01266486	Metformin	40 Participants with locally advanced breast cancer	IHC analysis of effects on phosphorylation of S6K, 4E-BP-1, and AMPK
Han et al. Phase II NCT01589367	Metformin vs. placebo in letrozole and no letrozole arms	208 Postmenopausal women with stage I/II ER-positive breast cancer	Clinical response rate at 24 weeks and comparison with RECIST 1.1 at baseline
Goodwin et al. (90) Phase III NCIC-MA.32, NCT01101438	Metformin vs. placebo	3,582 Non-diabetic participants with stage I/II node-positive or high-risk node-negative breast cancer	Invasive disease-free survival (IDFS)

DCIS, ductal carcinoma *in situ*; HR, hazard ratio; ER, estrogen receptor; HOMA, homeostasis model assessment.

^a^ Insulin resistance: HOMA index > 2.8, fasting glucose (mmol/L) × insulin (mU/L)/22.5.

## Statins

Statins have been used as cholesterol-lowering drugs for over three decades with great success (Figure 1). Preclinical *in vitro* and *in vivo* studies have demonstrated that statins inhibit proliferation of breast cancer cells, particularly ER-negative breast cancer cells (94), and growth of tumors in ER-negative breast cancer in mice (95). In addition, several epidemiologic studies have shown treatment with statins is associated with a reduced risk of a number of cancers, including breast (96). However, other epidemiologic studies have produced conflicting results (97). One meta-analysis of 16 breast cancer studies identified no preventive efficacy of statins (98), while other studies demonstrated significant reductions in breast cancer risk (99).

A number of Phase II prevention trials investigating the effects of statins on breast tissue biomarkers are currently ongoing. These studies have already demonstrated reduced proliferation and increased apoptosis associated with short-term treatment with statins (100, 101). Collectively, the results of epidemiologic, preclinical, and clinical studies suggest that statins may prevent breast cancer development and support the need for further investigation of their potential for the prevention of breast cancer, particularly TNBC.

## PARP Inhibitors

Poly(ADP-ribose) polymerase is a BRCA1/2 mutation-dependent DNA repair enzyme (102), and cells with loss of function BRCA1/2 mutations become selectively sensitive to the inhibition of PARP, which impairs homologous recombination and results in the induction of apoptosis (103). Several studies have shown that the loss of function of BRCA makes the cells deficient in homologous recombination DNA repair and makes these cells sensitive to PARP inhibitors (104) (Figure 1).

Recent studies have demonstrated that many TNBCs are characterized by shared sporadic and BRCA-mutated tumor characteristics (BRCA-ness), which exhibit impaired homologous recombination (105). Through array comparative genomic hybridization (aCGH) analyses, Lips et al. (105) have shown that a BRCA1-like array pattern and methylation of the BRCA1 promoter are apparent in 66–69 and 27–37% of TNBC tumors, respectively.

Clinical and preclinical data now suggests that PARP represents an effective target for the treatment of TNBC. Initial results of a Phase I clinical trial demonstrated that treatment with the PARP inhibitor olaparib as a single agent or in combination with DNA-damage-inducing chemotherapeutic agents is well-tolerated and presents few side effects (106). In addition, a Phase II clinical trial comparing the effects of chemotherapy alone vs. chemotherapy and the PARP inhibitor iniparib in women with metastatic TNBC, showed significant improvement of progression-free and overall survival in patients receiving iniparib (107). These results have led to a wave of additional clinical trials investigating the activity of PARP inhibitors in breast cancer patients, and suggest a potential utility and tolerability of PARP inhibitors for the prevention of breast cancer.

Recent data from preclinical animal studies has now shown that BRCA1 deficient mice treated with oral PARP inhibitors exhibit significant delays in tumor development (Sporn and Liby, unpublished results presented at the AACR Frontiers in Cancer Prevention Research Meeting, 2010). While this demonstrates the potential usefulness of PARP inhibitors for the prevention of ER-negative breast cancer, future results from clinical trials investigating PARP inhibitors for the prevention of breast cancer will determine the applicability of preventive strategies targeting PARP in women at high-risk of TNBC.

## IGF, mTOR, and S6K Inhibitors

Recently, advances have been made in testing novel targeted drugs in preclinical models, including mammalian target of rapamycin (mTOR), PI3K, and IGF1R inhibitors (Figure 1). Results from studies of these inhibitors suggest that they may also prove to be effective agents for the prevention of breast cancer.

The IGF1 pathway has been shown to be critical for mammary gland development, and IGF1 inhibitors could prove useful for prevention of both ER-positive and ER-negative breast cancers (108, 109). Although many studies have demonstrated the efficacy of IGF1R inhibition for the treatment of breast cancer, little progress has been made in determining its effect for the prevention of breast cancer (see Table 3). However, development of IGF pathway inhibitors has been slowed by toxicity (hypoglycemia) of drugs targeting this important pathway.

The PI3K/AKT/mTOR signaling pathway plays a critical role in regulating angiogenesis, cell growth, and proliferation (110, 111). mTOR is a serine-threonine kinase aberrantly and constitutively activated in many breast tumors (112), and results in tumorigenesis, angiogenesis, estrogen independence, and drug resistance (71, 113–115). Currently, several clinical trials are in progress investigating the effect of mTOR inhibitors (including everolimus, ridaforolimus, sirolimus, and temsirolimus) on HER2-positive breast cancer (116). To date, results reported from these trials have been contradictory. Preliminary results from GeparQuinto trial (117, 118) did not show significant improvements in pathologic response rates in women treated with both paclitaxel and everolimus compared to paclitaxel treatment alone. Conversely, Baselga and Colleagues recently reported interim results from the BOLERO-2 trial, which show improved progression-free survival (HR 57%, 95% CI 0.35–0.54) associated with combined exemestane-everolimus treatment vs. those receiving only exemestane (119).

Several *in vitro* and *in vivo* preclinical studies have investigated the potential mTOR inhibitors in the prevention of breast cancer. Using a set of TNBC cell lines that represent the progression to breast cancer, it was determined that treatment with rapamycin produces a larger effect on benign and pre-malignant cells than on breast cancer cells (74). mTOR inhibitors have also been investigated in a number of preclinical breast cancer prevention studies using mouse models (see Table 3). deGraffenried et al. have shown *in vitro* and *in vivo* mTOR inhibition and restored tamoxifen sensitivity following treatment with the mTOR inhibitor rapamycin (120). Recently, Hursting and Colleagues reported that treatment with the mTOR inhibitor everolimus abolishes the tumorigenic effects associated with obesity, improves calorie restriction-mediated anticancer activity, and blocks mammary tumor development and mTOR activation (121). These and other positive results suggest mTOR inhibitors may prove to be particularly useful cancer prevention agents in women at high-risk of breast cancer.

Critical downstream kinases in the mTOR/S6K signaling pathway are the ribosomal S6 kinases, with p70S6K (S6K1) functioning as the main family member downstream of mTOR. p70S6K is activated by a variety of signals and induces cell growth, proliferation, cell survival, and other oncogenic processes. p70S6K is frequently amplified and overexpressed in cancer cells, and its hyperactivation has been associated with the frequently mutated tumor suppressor LKB1 (STK11) (122). siRNA or chemical inhibition (with PF4708671) of S6K1 enhances cell death in glucose deprivation conditions (123). Becker et al. recently reported that inhibition of p70S6K inhibits IGF-induced ER activation, p70S6K binding, and ER target gene activation (124). Another S6 kinase family member, RSK (p90S6K), has been shown to regulate TNBC growth and survival through the phosphorylation and activation of Y-box binding protein-1 (YB-1) (125, 126). Although not tested in the prevention of breast cancer yet, the recent findings on mTOR/S6K pathway make it a promising target for the prevention of breast cancer.

## Natural Products

Increased understanding of the correlation between a healthy diet and reduced cancer incidence of a variety of cancer types, has led many researchers to focus on natural products for the prevention of cancer. According to the National Health and Nutrition Examination Survey (NHANES) over 49% of adults in the U.S. took a dietary supplement between 2007 and 2010, of whom 32% took dietary supplements containing an antioxidant (e.g., vitamins C and E, β-carotene, resveratrol, flavonoids, or isoflavones) (127). Of those adults taking supplements, NHANES data show that slightly less than one quarter take them upon the recommendation of a health care provider.

A recent meta-analysis of over 5,000 breast cancer cases, reported an inverse association between green tea consumption and breast cancer incidence (128). In 2012, our group reported results from a Phase Ib clinical trial using green tea epigallocatechin gallate (EGCG) over a 6-month period, which was conducted to determine the maximum tolerated dose (MTD) (129). During the treatment period no changes in breast tissue proliferation were observed. Overall, the agent was well-tolerated, with toxicity data establishing a 600-mg twice daily MTD for Poly E (EGCG). A Phase II trial testing the cancer preventive effects of 1 year of EGCG in postmenopausal women with high mammographic is currently ongoing.

Resveratrol (3,5,4′-trihydroxy-trans-stilbene) is a non-flavonoid polyphenol present in the skins of red grapes, mulberries, and other plants. Resveratrol has been reported to have a wide range of health benefits through many different mechanisms of action. Recently, resveratrol has been implicated in glucose metabolism, and has been shown to cause growth inhibition and apoptosis in cancer cells (130, 131). Although resveratrol is well-known for its health benefits, its role as a cancer preventive agent is not yet well-accepted. Further human studies need to be performed to establish the appropriate dose and treatment duration.

Many other vitamins and natural products are being tested as cancer preventive agents, and specifically for breast cancer prevention. Studies of these agents have been previously reviewed (132, 133). However, despite strong interest in using natural products that have little known toxicities, none of these dietary agents have yet been shown to have cancer preventive activity.

Currently, no FDA-approved drugs are available for targeted breast cancer prevention in women at high-risk of ER-negative breast cancer. However, a number of promising agents are being investigated in preclinical and clinical trials, and encompass a wide range of targeted strategies, including preventive therapy with drugs such as retinoids, COX-2 inhibitors, metformin, statins, PARP inhibitors, and signal transduction (IGF, mTOR, S6K) inhibitors, as well as natural products, such as EGCG and resveratrol. In addition, vaccines and behavioral strategies are being tested for breast cancer prevention. The potential efficacy of many of these preventive strategies for the prevention of ER-negative, and particularly triple-negative, breast cancer will be determined in the near future. The identification and development of multiple specific agents targeting critical oncogenic pathways will be essential for effective prevention of breast cancer in women at high risk of TNBC.

## Conclusion and Future Perspective

Great progress has been made in the treatment and prevention of ER-positive breast cancer. Most recently, several breast cancer prevention trials targeting HER2 have demonstrated the preventive efficacy of HER2-targeting drugs. However, the identification and develop of effective and safe targeted therapies for the prevention of TNBC remains challenging. This difficulty is exacerbated by the inherent heterogeneity of TNBC tumors, and underlines the necessity for subtype-specific multi-targeted approaches. Such combined strategies are critical for effective treatment and prevention of women with or at high risk of TNBC due to both the diverse signaling pathways driving the different molecular subtypes, as well as the presence of cancer stem cells, which are difficult to eradicate and are overrepresented in women with TNBC.

Successful clinical breast cancer prevention trials hinge upon the ability to identify high-risk individuals by breast cancer subtype, who therefore carry a high-potential benefit for the agent(s) being tested in the study. However, educating women at risk for breast cancer about the risks and benefits of the different cancer preventive drugs currently under investigation has been and remains challenging. Although a large population of women qualifies for these therapies, very few enroll in clinical breast cancer prevention trials. As the majority of these drugs is well-tolerated by breast cancer patients and are typically associated with only minor side effects, the consent, and participation of healthy high-risk women in clinical breast cancer prevention trials represents a major hurdle to the development of novel therapeutic strategies. Effective reduction of breast cancer incidence in the years to come, particularly ER-negative and TNBC, ultimately depends upon the identification of novel targets, the development of non-toxic drugs that effectively interrupt the activity of those targets, and the delivery of these preventive therapies to women at high-risk of breast cancers characterized by the targeted agents.

## Author Contributions

Literature review: Petra den Hollander, Michelle I. Savage, and Powel H. Brown writing, review, and/or revision: Petra den Hollander, Michelle I. Savage, and Powel H. Brown.

## Conflict of Interest Statement

Powel H. Brown is on the Scientific Advisory Board of Susan G. Komen for the Cure. All remaining authors declare no actual, potential, or perceived conflict of interest that would prejudice the impartiality of this article.
